# Male Circumcision and HIV Control in Africa

**DOI:** 10.1371/journal.pmed.0030078

**Published:** 2006-01-31

**Authors:** Michel Garenne

**Affiliations:** **1**Institut PasteurParisFrance

In a recent article, Auvert and colleagues present the results of their randomized controlled trial on male circumcision to prevent HIV transmission [ 1]. They conclude that male circumcision reduced the risk of HIV infection by some 60% (95% confidence interval, 32%–76%). The trial was certainly well conducted, and it nicely confirmed observational studies, which came to the same conclusion [ 2]. However, a number of their concluding statements deserve a comment.

Auvert and colleagues claim a “degree of protection equivalent to a vaccine of high efficacy” [ 1]. This is obviously overstated. A vaccine of high efficacy is expected to offer long-term protection of 95% or above. Smallpox was eradicated with such a highly efficient vaccine. If control of tetanus, measles, and poliomyelitis has been largely achieved in the world, it has been a result of high-efficacy vaccines. Furthermore, the analogy with vaccines appears misleading. A 96%-efficient measles vaccine means that 96% of vaccinated persons exposed to measles are indeed protected against infection. Protection lasts for many years, and revaccination permits dealing with loss of immunity over time. What Auvert and colleagues show is different: they show a 60% reduction in disease incidence over an 18-month period among circumcised men compared with uncircumcised men with similar exposure. To our knowledge, this does not mean that those men are really “protected” against HIV, especially in the case of repeated exposure. It simply means “reduced risk,” or reduced probability of contamination.

A closer analogy of the “reduced risk” offered by male circumcision is that offered by contraception. Modern and efficacious methods such as hormonal contraceptives (pill, injectables, implants) or intra-uterine devices (IUDs) do offer high protection, usually 99% or above for women who are exposed repeatedly (every month) to risk of pregnancy. Highly efficacious methods do protect these women against unwanted pregnancy. On the contrary, a less efficacious method such as rhythm method (periodic abstinence) reduces fecundity by some 50%, but offers little protection against unwanted pregnancy. Even though women using consistent rhythm methods will have a lower number of pregnancies over their lifetime than women who use no contraceptive methods at all, they will be unlikely to achieve their desired family size, as could women using highly effective methods.

Similarly, for persons who are highly exposed to risk of HIV infection, as are the young men of South Africa, a 60% reduction in annual risk will ultimately protect only a smaller proportion. Basic probability calculations show that in discordant couples exposed for 30 years, some 74% will contract the HIV virus if circumcised, compared with 97% if uncircumcised (with incidence of 11% per year)—a small reduction indeed if compared with a highly efficacious vaccine (comparable figures would be 4% versus 97% for children vaccinated against measles who are exposed between 1 and 15 years of age).

One could argue that the population effect could exceed the individual risk for a variety of reasons ranging from herd immunity to prevention of other sexually transmitted diseases (STIs). If all men are circumcised, then prevalence among women will be lower, and men will have lower risk of being exposed and infected. However, several natural experiments do not confirm this argument. For instance, Tanzania has some 110 ethnic groups, some groups using universal male circumcision, others not circumcising. After controlling for urbanization, there was no difference in male HIV prevalence between the two groups: in urban areas, HIV seroprevalence was 9.5% in circumcised groups and 9.7% in uncircumcised groups, and conversely, 4.6% and 5.2%, respectively, in rural areas—none of the differences being significant [ 3]. In South Africa, the KwaZulu-Natal province, where few are circumcised, has a higher HIV seroprevalence than other provinces, reaching 37% among antenatal clinic attendants in 2003. But, in the Eastern Cape, where circumcision is the rule, the dynamics of the epidemic are almost the same, simply lagging a few years behind, increasing from 4.5% in 1994 to 27% in 2003. Finally, it was argued that the large epidemic in Abidjan, Côte d'Ivoire, and surrounding areas in the late 1980s was largely due to the lack of male circumcision of the local ethnic groups. This, however, did not impede the rapid increase in HIV infection among migrant workers from Burkina Faso and Mali living in Abidjan, who were circumcised.

For highly exposed men, such as men living in southern Africa, the choice is either using condoms consistently, with extremely low risk of becoming infected, or being circumcised, with relatively high risk of becoming infected. This is quite similar to women's choice to either use a highly efficacious contraceptive method or use a folk method. Some women make the second choice for religious reasons, with the obvious consequences. Is there a rationale for promoting the idea of circumcision when better choices are available? Regular condom use was found to be protective at the individual level and also effective for stopping HIV epidemics, as in Thailand [ 4, 5].

Concluding that “male circumcision should be regarded as an important public health intervention for preventing the spread of HIV” [ 1] appears overstated. Even though large-scale male circumcision could avert a number of HIV infections, theoretical calculations and empirical evidence show that it is unlikely to have a major public health impact, apart from the fact that achieving universal male circumcision is likely to be more difficult than universal vaccination coverage or universal contraceptive use.
